# A systematic review of newborn and childhood hearing screening around the world: comparison and quality assessment of guidelines

**DOI:** 10.1186/s12887-022-03234-0

**Published:** 2022-03-29

**Authors:** Cheng Wen, Xuelei Zhao, Yue Li, Yiding Yu, Xiaohua Cheng, Xiaohong Li, Kui Deng, Xuelian Yuan, Lihui Huang

**Affiliations:** 1 Key Laboratory of Otolaryngology, Head and Neck Surgery, Ministry of Education, Beijing Institute of Otolaryngology, Beijing Tongren Hospital, Capital Medical University, No. 17 Hougou Lane, Chongnei Street, Beijing, 100005 China; 2 National Center for Birth Defect Monitoring of China, West China Second University Hospital, Sichuan University, Chengdu, 610041 Sichuan China

**Keywords:** Hearing screening, Newborn, Childhood, Guidelines, Systematic review

## Abstract

**Background:**

This study aimed to assess the quality of global guidelines or consensus statements for newborn and childhood hearing screening, as well as to compare various guidelines between other countries and China.

**Methods:**

A PROSPERO registered systematic review (number CRD42021242198) was conducted. Multiple electronic databases and government websites including PubMed, EMBASE, Web of Science, CENTRAL, Cochrane Library, and BMJ Best Practice were searched from inception until May 2021. The latest national and international guidelines, consensus statements, technical specifications, and recommendations regarding newborn or childhood hearing screening that were published in Chinese or English medical journals or elsewhere with the full version available online. The following information was extracted independently by two reviewers for comparative analysis: titles, authors, publication year, country, the source organization, and main key recommendations using systems for assigning the level of evidence and strength of recommendations. The quality of the guidelines was assessed by three independent reviewers using the Appraisal of Guidelines for Research and Evaluation, 2nd edition. Intraclass correlation coefficients (ICCs) were calculated to assess among-reviewer agreement.

**Results:**

We assessed 15 newborn and 6 childhood hearing screening guidelines, respectively. Most newborn guidelines recommend the 1–3-6 guidelines and pre-discharge screening; however, the specific screening times differ. 93.33% of newborn hearing guidelines recommend “primary screening-re-screening-diagnosis-intervention” for well-babies while 73.33% of the guidelines recommend "initial screening-diagnosis-intervention" for newborns in neonatal intensive care unit (NICU); 33.33% of the newborn hearing guidelines recommended initial screening coverage of > 95% while 46.66% did not mention it. Further, 26.66% of the newborn hearing guidelines recommended a referral rate to diagnosis within 4% while 60% did not mention it. Regarding childhood hearing screening guidelines, the screening populations differed across guidelines (age range: 0–9 years); most guidelines recommend pediatric hearing screening for all preschoolers. Only 50% of the guidelines specify screening and re-screening techniques, including pure-tone hearing screening, OAE, tympanometry, and others. The “Clarity of Presentation” domain achieved the highest mean score, and the lowest was “Editorial Independence” both in newborn and childhood guidelines. Overall score of newborn hearing screening guidelines ranged from 3 (2018 Europe) to 7 (2019 America), with an average score of 5.33. Average score of childhood hearing screening guidelines was 4.78, with the score ranging from 4 (2017 England, 2012 Europe, 2016 WHO) to 6.67 (2011 America). ICC analysis revealed excellent agreement across 21 guidelines (> 0.75).

**Conclusions:**

These findings indicated newborn hearing screening guidelines had superior quality over childhood ones. Comparative analysis suggested that recommendations of the Chinese newborn and pediatric hearing screening protocols are consistent with the mainstream international opinion. Moreover, this analysis demonstrated that “Editorial Independence” and “Stakeholder Involvement” have the greatest opportunities for improvement. These results may help to advance the quality of hearing screening guidelines in clinical practice and guide evidence-based updates.

## Background

Hearing loss is among the most common congenital disabilities worldwide. The World Report on Hearing published by the World Health Organization (WHO) indicates that > 1.5 billion people currently experience some degree of hearing loss, which could grow to 2.5 billion by 2050 [1]. The WHO estimates that over 400 million people, including 34 million children, live with disabling hearing loss, which affects their health and quality of life [1]. The global prevalence of moderate-to-severe hearing loss increases with age, which increases from 0.2% in early neonates to 1.5% in children aged 5–9 years [1]. The impact of hearing loss on children is dependent on age at onset and severity; moreover, there is a need for clinical and rehabilitative measures [2]. Delaying hearing tests negatively affects growing children in terms of delayed language acquisition, speech development, literacy, and social skills. According to WHO, early detection through universal newborn hearing screening (UNHS) could reduce the burden of hearing loss.

UNHS is standard in numerous countries, including the US and UK, and allows early detection, diagnosis, and interventions. Both US and UK implemented screening guidelines for screening management and improving screening quality early in 1990s [3, 4]. The UNHS program has been implemented in China for more than 20 years and also contributed to early detection, diagnosis, and interventions, with good social results [5, 6]. In 2004, the former Ministry of Health enacted “Technical Specifications for Newborn Hearing Screening” and promulgated the “Technical Specification for Newborn Hearing Screening (2010 Edition)” in 2010. WHO has paid increasing attention to the Chinese UNHS program. Wilson et al. reported that UNHS program is effective in high-income countries, including China, to identify serious problems promptly [7]. Regarding guidelines for newborn hearing screening issued worldwide, there is a need to determine their quality, the consistency of relevant information, and the utility of analyzing them. Physiological measures, including otoacoustic emissions (OAE) and automated auditory brainstem response (AABR), can be used to screen newborns and infants for hearing loss. Both can be easily applied and have been successfully used for UNHS programs; however, they have important differences. OAE measurements are obtained from the ear canal using a sensitive microphone within a probe assembly for recording cochlear responses to acoustic stimuli [4]. Accordingly, OAEs reflect the status of the peripheral auditory system extending to the cochlear outer hair cells; moreover, it is easy, fast, sensitive, and inexpensive. Contrastingly, auditory brainstem response (ABR) measurements are obtained from surface electrodes that record neural activity in the cochlea, auditory nerve, and brainstem in response to acoustic stimuli delivered through an earphone. AABR measurements reflect the status of the peripheral auditory system, the eighth nerve, and the brainstem auditory pathway. Moreover, they allow effective screening for auditory neuropathy. However, they are time-consuming and costly [4].

Additionally, there are several risk factors for late-onset permanent hearing loss during pre-school years, as demonstrated by the 2007 Joint Committee on Infant Hearing statement [4]. Delayed- or late-onset hearing loss involves normal auditory function at birth followed by the onset of auditory dysfunction and associated hearing loss during infancy or early childhood. Depending on the etiology, hearing loss may be unilateral or bilateral; further, it may affect any frequency. Hearing loss often gradually worsens during early childhood and even into school-age years [8]. Up to 50% of 9‐year‐old children with educationally significant hearing loss have undergone newborn hearing screening [9]. Approximately 9–10 per 1000 children present identifiable permanent unilateral or bilateral hearing loss by school‐age [10]. According to the World Hearing Report published in 2021, late-onset or progressive hearing loss related to these conditions is often missed during early childhood screening [1]. In 2011, the American Academy of Audiology issued Childhood Hearing Screening Guidelines for developing evidence-based recommendations for screening hearing in 6-month-old children throughout high school [11]. To protect and promote children's hearing and speech development, as well as reduce hearing and speech disabilities in children, the National Health and Family Planning Commission of the People's Republic of China promulgated the Technical Specification for Children's Ear and Hearing Care in 2013 [12]. There is a need to explore the publication, quality, and recommendations of children hearing screening guidelines worldwide. The age ranges used in the World Report on Hearing were: perinatal period, 0–4 years; childhood and adolescence, 5–17 years [1]. In this article, childhood hearing screening is primarily distinguished from newborn hearing screening. Most guidelines recommend newborn hearing screening within the first month of life and pediatric hearing screening for all preschool and school-age children, with minor differences in details between guidelines.

The Appraisal of Guidelines for Research and Evaluation (AGREE II) refers to a set of tools for methodically assessing the quality of clinical practice guidelines and consensus statements [13]. It contains 23 items that assess scope and purpose, stakeholder involvement, rigor of development, clarity of presentation, applicability, and editorial independence. It has been widely applied in different areas, including newborn hearing screening, chronic sinusitis, head and neck cancer, and the detection and management of otitis media. In 2021, Chorath et al. identified and evaluated 12 guidelines for the detection and management of neonatal hearing loss, demonstrating that the ‘Rigor of Development’ and ‘Editorial Independence’ have the greatest opportunities for improvement [14]. However, the 12 newborn hearing screening guidelines included in the study did not include the Chinese guidelines and the study did not compare the screening protocols among the guidelines. Accordingly, our systematic review assessed not only the quality of global newborn hearing screening guidelines and consensus statements, but also the childhood hearing screening guidelines, and analyzed the characteristics of various guidelines between other countries and China.

## Methods

This systematic review was conducted following the Cochrane methodology and the latest preferred reporting items for systematic reviews and meta-analyses [15, 16]. Since this was a systematic literature review, ethics approval was not required. A protocol exists for the systematic review and the registered PROSPERO number is CRD42021242198.

### Data sources and search strategy

We queried multiple peer-reviewed databases to identify relevant articles. The English databases included PubMed, EMBASE, Web of Science, CENTRAL, Biomed Central, Cochrane Library, BMJ Best Practice, Guidelines International Network, National Institute for Health and Clinical Excellence, National Guideline Clearinghouse, MEDLINE, Scottish Intercollegiate Network and Google Scholar. The Chinese databases included the China National Knowledge Infrastructure, Wan Fang Data Knowledge Service Platform, Chinese Biomedical Literature, and China Science and Technology Journal Database (VIP). Since the database queries only retrieved journal-published guidelines and several guidelines are only published on their websites, we used Google and Baidu to search for 10 relevant foreign government websites and two Chinese government websites. All databases and government websites were searched from inception until May 2021. Table 1 summarizes the sample search strategy based on the indexing systems.

**Table 1:** Sample search strategy on PubMed database

Database	PubMed
Date	09/05/2021
strategy	#1 AND #2 AND #3 AND #4 AND #5 AND #6 AND #7
#1	(sensorineural hearing loss[MeSH Terms]) OR (sensorineural hearing loss[Title]) OR (Hearing loss[Title]) OR (Hearing loss[MeSH Terms]) OR (Hearing Impairment[MeSH Terms]) OR (Hearing Impairment[Title]) OR (Hearing Impairments[Title]) OR (deafness[Title]) OR (deafness[MeSH Terms]) OR (hearing disorders[MeSH Terms]) OR (Hearing Disorders[Title]) OR (Hearing Disorder[Title]) OR (congenital hearing loss[Title]) OR (neonatal hearing loss[Title]) OR (newborn hearing loss[Title])
#2	(neonatal screening[Title]) OR (newborn screening[Title]) OR (selective screening[Title]) OR (risk factor screening[Title]) OR (screenings[Title]) OR (mass screening[Title]) OR (universal screening[Title]) OR (newborn hearing screening[Title]) OR (universal newborn hearing screening[Title]) OR (Early Detection of hearing loss[Title]) OR (early detection deafness[Title]) OR (early hearing loss diagnosis[Title]) OR (early deafness diagnosis[Title]) OR (preschool screening[Title]) OR (pre-school screening[Title]) OR (child screening[Title]) OR (children screening[Title]) OR (childhood screening[Title]) OR (pediatric screening[Title])
#3	(guideline[Publication Type]) OR (guidelines[Title]) OR (guideline[Title]) OR(Practice Guideline[Publication Type]) OR (Practice Guideline[Title]) OR (Practice Guidelines[Title]) OR (Guidelines as Topic[MeSH Terms]) OR (Health Planning Guidelines[MeSH Terms]) OR (Health Planning Guidelines[Title]) OR (Health Planning Guideline[Title]) OR (guidance[Title]) OR (consensus[MeSH Terms]) OR (Standard of Care[MeSH Terms]) OR (consensus[Title]) OR (criterion[Title]) OR (criterions[Title]) OR (recommendation[Title]) OR (recommendations[Title]) OR (standard[Title]) OR (standards[Title]) OR (strategy[Title]) OR (strategies[Title]) OR (criteria[Title]) OR (manual[Title]) OR (guidebook[Title]) OR (guidebooks[Title]) OR (guide[Title]) OR (guides[Title]) OR (handbook[Title]) OR (handbooks[Title]) OR (references[Title]) OR (reference[Title]) OR (referral[Title]) OR (referrals[Title])

### Inclusion and exclusion criteria

We included the latest national and international guidelines, consensus statements, technical specifications, and recommendations regarding newborn or childhood hearing screening that were published in Chinese or English medical journals or elsewhere with the full version available online. We excluded repetitive literature, guidelines without full text.

### Data extraction and management

Two reviewers independently reviewed the titles, keywords, and abstracts; subsequently, they included articles based on the relevance criteria. Discrepancies were resolved through consensus or consulting with a third reviewer. The following information was extracted: titles, authors, publication year, country, the source organization, and main key recommendations using systems for assigning the level of evidence and strength of recommendations.

### Quality appraisal

Quality assessment was conducted using the AGREE II instrument, which provides a systematic framework for assessing the methodological rigor of guideline quality, as well as a methodological strategy for developing guidelines [13]. As shown in Table 2, the AGREE II instrument includes 23 items for assessing the aforementioned six domains. Items are rated on a 7-point scale ranging from 1 (absence of items) to 7 (exceptional quality of item). Three trained appraisers with a background in audiology studies and experience with hearing screenings independently appraised each item using the AGREE II. Between-reviewer disagreements were resolved through consensus or consultation with an independent expert adjudicator. Domain scores were calculated by summing the item scores within each domain for each reviewer as follows: scaled domain score = (obtained score-minimum possible score) / (maximum possible score-minimum possible score) × 100%. The overall scores for each guideline were calculated and reported as means.

**Table 2:** AGREE II instrument

Domain	Number	Item
DOMAIN 1. SCOPE AND PURPOSE	1	The overall objective(s) of the guideline is (are) specifically described
	2	The health question(s) covered by the guideline is (are) specifically described
	3	The population (patients, public, etc.) to whom the guideline is meant to apply is specifically described
DOMAIN 2. STAKEHOLDER INVOLVEMENT	4	The guideline development group includes individuals from all relevant professional groups
	5	The views and preferences of the target population (patients, public, etc.) have been sought
	6	The target users of the guideline are clearly defined
DOMAIN 3. RIGOR OF DEVELOPMENT	7	Systematic methods were used to search for evidence
	8	The criteria for selecting the evidence are clearly described
	9	The criteria for selecting the evidence are clearly described
	10	The methods for formulating the recommendations are clearly described
	11	The health benefits, side effects, and risks have been considered in formulating the recommendations
	12	There is an explicit link between the recommendations and the supporting evidence
	13	The guideline has been externally reviewed by experts prior to its publication
	14	A procedure for updating the guideline is provided
DOMAIN 4. CLARITY OF PRESENTATION	15	The recommendations are specific and unambiguous
	16	The different options for management of the condition or health issue are clearly presented
	17	Key recommendations are easily identifiable
DOMAIN 5. APPLICABILITY	18	The guideline describes facilitators and barriers to its application
	19	The guideline provides advice and/or tools on how the recommendations can be put into practice
	20	The potential resource implications of applying the recommendations have been considered
	21	The guideline presents monitoring and/or auditing criteria
DOMAIN 6. EDITORIAL INDEPENDENCE	22	The views of the funding body have not influenced the content of the guideline
	23	Competing interests of guideline development group members have been recorded and addressed
OVERALL GUIDELINE ASSESSMENT	1	Rate the overall quality of this guideline
	2	I would recommend this guideline for use

*AGREE II* Appraisal of Guidelines for Research & Evaluation

### Data analysis

Descriptive statistical analysis was conducted; moreover, the between-reviewer agreement was assessed using two-way, random, single unit, absolute agreement intra-class correlation coefficients (ICC) [17]. The degree of reviewer agreement was categorized based on Cicchetti (1994) as follows: ICC < 0.40, poor; 0.40–0.59, moderate; 0.60–0.74, good; 0.75–1.00, excellent [18].

## Results

**Fig. 1:**
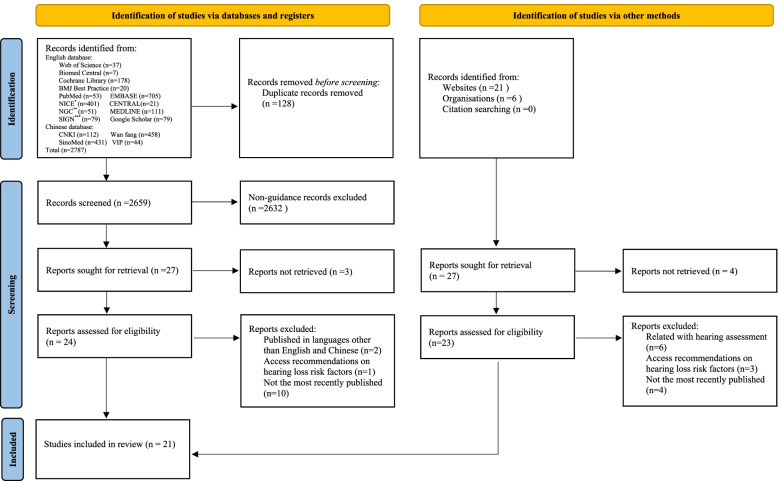
Flow diagram for identification of clinical practice guidelines and consensus statement. * NICE: National Institute for Health and Clinical Excellence; **NGC: National Guideline Clearinghouse; ***SIGN: Scottish Intercollegiate Network

Our electronic search yielded 2814 citations. Based on the inclusion/exclusion criteria, 21 articles were included (Fig. 1); among them, 15 were newborn hearing screening guidelines [3, 19–32], and six were childhood hearing screening guidelines [8, 11, 12, 33–35], respectively.

### Newborn hearing screening guidelines

#### Characteristics of newborn hearing screening guidelines

Table 3 provides specific details regarding the country or region, developer, year, title, screening protocols, initial screening coverage, rate of referral to diagnosis, diagnosis time, intervention time, and follow-up duration for newborns at risk.

**Table 3:** Characteristics and key recommendations of newborn hearing screening guidelines included in the study

No	Country/region	Developer	Year	Title	Well-babies				NICU newborns		Initial screening coverage (%)	Referral rate to diagnosis (%)	Diagnosis time	Intervention time	Follow-up time for risk newborns
					First screening time	First screening technology	Rescreening time	Rescreening technology	With/without rescreening	Screening technology					
1	America [19]	The Joint Committee on Infant Hearing	2019	Year 2019 Position Statement: Principles and Guidelines for Early Hearing Detection and Intervention Programs	Before discharge	OAE/AABR	Within 1 month	OAE/ AABR	Without	AABR	> 95	/	Within 3 months	Within 6 months	Age of 9 months
2	Europe [20]	European Standards of Care for Newborn Health	2018	Hearing screening	During the first weeks of life	AABR	Within 1 month	AABR	Without	AABR	/	/	Within 3 months	Within 6 months	/
3	Germany [21]	Matula P et al	2018	The Newborn Hearing Screening Program in Germany	On the second or third day of life before discharge	OAE/AABR	/	AABR	Without	AABR	> 95	< 4	Within 3 months	Within 6 months	
4	South Africa [22]	The Health Professions Council of South Africa	2018	Professional Board for Speech Language and Hearing Professions: Early Hearing Detection and Intervention (EHDI)	Before 1 month of age and within 6 weeks of age	OAE	Within 1 month after discharge, approximately 2.5 months of age	OAE	With	AABR	> 95	< 5	Before 3 months of age and within 4 months of age	Before 6 months of age and within 8 months of age	Age of 9 months
5	International [23]	Farinetti A et al	2018	International Consensus (ICON) on Audiological Assessment of Hearing Loss in Children	Before discharge	OAE	Within 1 month	OAE/AABR	/	AABR	> 95	/	Within 3 months	Within 6 months	/
6	India [24]	Indian Academy of Pediatrics	2017	Consensus Statement of the Indian Academy of Pediatrics on Newborn Hearing Screening	72 h after birth or on the day of discharge	OAE	Four weeks after first screening, or at 6 weeks on the first immunization visit	OAE	Without	ABR	/	/	Within 3 months	Within 6 months	/
7	Italy [25]	Berrettini S et al	2017	Newborn hearing screening protocol in the Tuscany region	24 h after birth and before discharge	OAE	Within 1 month	AABR	Without	OAE + AABR	> 98	/	Within 3 months	Within 6 months	Age of a year
8	England [3]	Public Health England	2016	NHS Newborn Hearing Screening Program: Standards 2016 to 2017	72 h after birth to 10 days of age	OAE	Within 4–5 weeks of age	/	/	AABR	97 ~ 99.5	2.5–3	Within 4 weeks of screen completion	Within 4 weeks of screen completion or by 44 weeks gestational age	/
9	New Zealand [26]	Ministry of Health	2016	Universal Newborn Hearing Screening and Early Intervention Program: National Policy and Quality Standards	/	OAE/AABR	Within 1 month	OAE/ AABR	Without	AABR	/	/	Within 3 months	Within 6 months	Age of 18 months
10	International [27]	IPOG	2016	IPOG Consensus Recommendations: Hearing loss in the Pediatric Patient	/	OAE/AABR	/	AABR	Without	OAE + AABR	/	/	/	/	/
11	Australia [28]	Neonatal Hearing Screening Working Group	2013	National Framework for Neonatal Hearing Screening	24–72 h after birth and before discharge	OAE/AABR	2 weeks after first screening, within 1 month of age	OAE/ AABR	Without	OAE + AABR	> 97	< 4	Two weeks after first screening within 3 months of corrected age	Within 3 months and no later than 6 months of age	Age of 1 year
12	Canada [29]	Canadian Paediatric Society	2011	Universal newborn hearing screening	/	OAE	Within 1 month	AABR	Without	AABR	/	2–4	Within 3 months	Within 6 months	/
13	China [30]	Ministry of Health of the People's Republic of China	2010	Technical specifications for newborn hearing screening	48 h after birth and before discharge	OAE/AABR	Within 42 days of age	OAE/AABR	Without	AABR	/	/	Within 3 months	Within 6 months	At least once every 6 months until the age of 3 years
14	Spain [31]	CODEPEH	2010	Early Hearing Detection and Intervention: 2010 CODEPEH Recommendation	Before the first month of life	OAE/AABR	Within 1 month	OAE/AABR	Without	OAE + AABR/AABR	> 95	< 4	Within 3 months	Within 6 months	24–30 months of age
15	WHO [32]	WHO	2010	Newborn and infant hearing screening: current issues and guiding principles for action	/	OAE/AABR/others	Within 1 month	OAE/AABR/others	/	AABR	/	/	Within 3 months	Within 6 months	/

*OAE* Otoacoustic emissions, *AABR* Automated auditory brainstem response, *IPOG* International Pediatric Otolaryngology Group, *CODEPEH* Commission for the Early Detection of Hypoacusis, *WHO* World Health Organization

#### General information of newborn hearing screening guidelines

Table 3 presents 15 guidelines from 15 countries or organizations published between 2010 and 2019. Three guidelines were developed by international committees: the International Consensus on Audiological Assessment of Hearing Loss in Children (ICON), International Pediatric Otolaryngology Group (IPOG), and WHO. Twelve guidelines were developed by expert groups, including the Joint Committee on Infant Hearing of America, European Standards of Care for Newborn Health, the Health Professions Council of South Africa, Indian Academy of Pediatrics, Public Health England, Ministry of Health of New Zealand, Neonatal Hearing Screening Working Group of Australia, Canadian Pediatric Society, Ministry of Health of the People's Republic of China, the Commission for the Early Detection of Hypoacusis of Spain, University Hospital Muenster and University Hospital of Pisa.

Sixty percent of the guidelines, which were from America, Europe, Germany, South Africa, England, Australia, Canada, China, and WHO, stipulate that parents should be informed about the medical background of the UNHS program and the screening procedure. The remaining guidelines were from ICON, India, Italy, New Zealand, IPOG, and CODEPEH.

#### Screening principle and time of newborn hearing screening guidelines

As shown in Table 3, 53.33% (8/15) guidelines recommend the 1–3-6 principles, with all infants being required to undergo hearing screening within the age of 1 month. However, 2010 China guideline recommends screening within 42 days after birth. Moreover, 11 (73.33%) guidelines, including the 2010 China guideline, recommend that individuals who have not undergone prompt screening receive a diagnostic audiological evaluation within 3 months after birth. Moreover, they recommend prompt provision of audiological, medical, and educational services to infants diagnosed with hearing loss within 6 months after birth.

Seven guidelines recommend initial screening before discharge; however, there are differences in the specific screening times. The recommended times for initial screening in guidelines from Europe, Germany, UK, Australia, and China are within 1 week, 2–3 days, 72 h, 72 h, 24–72 h, and 48 h after birth, respectively. Additionally, 4 (26.67%) guidelines did not mention the specific initial screening time. Regarding the rescreening time, 9 (60%) of the guidelines recommend completion within 1 month, 4 (26.67%) have different recommendations, and 2 (13.33%) do not mention it. The follow-up time for newborns at risk widely ranged from the age of 9 months (America, South Africa) to 36 months (China). It is worth noting that the 2019 America guideline mentions that programs meeting current targets might consider setting a new target of 1–2-3 months (screening completed by one month of age, audiologic diagnosis completed by two months of age, and early intervention initiated no later than three months of age).

#### Screening protocols of newborn hearing screening guidelines

##### (1) Well-babies

As shown in Table 3, 14 (93.33%) guidelines recommend “primary screening-rescreening-diagnosis-intervention” as the process for hearing screening for well-babies, with the WHO guidelines not mentioning this.

Table 3 summarizes the initial screening technologies for well-babies. We found that 6 (40%), 1 (6.67%), and 7 (46.66%) guidelines recommend OAE only, AABR only, and both, respectively. The 2010 WHO guideline recommends OAE or AABR as the most accurate technologies with universal feasibility; moreover, other methods, including family questionnaires and behavioral measures, can be used depending on the circumstances.

Regarding rescreening technologies for well-babies, 6 guidelines (40%), including China’s technical specifications, recommend OAE or AABR; 5 (33.33%) recommend only AABR; 13.33% (2/15) recommend only OAE; 1 (6.67%) recommends OAE, AABR, or other technologies; and 1 (6.67%) did not mention it.

##### (2) NICU newborns

Eleven (73.33%) guidelines, including China’s technical specifications, recommend "initial screening-diagnosis-intervention" as the hearing screening protocol for newborns in neonatal intensive care unit (NICU). Briefly, rescreening is not recommended for NICU newborns who fail initial screening; instead, they are directly referred to the hearing diagnostic center for hearing diagnosis. Moreover, one (6.67%) guideline recommends that NICU newborns who fail initial screening should undergo rescreening before hearing diagnosis while three (20%) guidelines did not mention relevant information.

Regarding the screening technology for NICU newborns, 11 (73.33%) guidelines, including China’s technical specifications, recommend AABR; 2 (13.33%) recommend combining OAE and AABR; 1 (6.67%) recommends ABR; and 1 (6.67%) recommends combining OAE and AABR or only AABR.

#### Screening quality indicators of newborn hearing screening guidelines

Common quality control indicators include initial screening coverage and referral rates for diagnosis. Five (33.33%), two (13.33%), and one (6.67%) guideline recommended initial screening coverage of > 95%, > 97%, and > 98%, respectively. Furthermore, 4 (26.66%), 1 (6.67%), and 1 (6.67%) guideline recommended referral rates to diagnosis within 4%, 5%, and 3%, respectively. Seven (46.67%) and nine (60%) guidelines did not mention initial screening coverage and the referral rate to diagnosis, respectively. China’s technical specifications did not mention either.

### Childhood hearing screening guidelines

#### Characteristics of childhood hearing screening guidelines

Table 4 provides specific details regarding the country or region, developer year, title, screening populations, screening technology, information about rescreening or diagnosis, and key recommendations.

**Table 4:** Characteristics and key recommendations of the included childhood hearing screening guidelines

No	Country/region	Developer	Year	Title	Screening populations	Screening technology	Rescreening/diagnosis	Key recommendations
1	England [33]	Audiology and Health	2017	Early identification of deafness in childhood (following newborn hearing screening) position statement	4–7 years of age	/	/	1. Data should be collected locally and nationally 2. Include local audit and clinical governance arrangements 3. Obtain parental consent before hearing screening
2	International [34]	World Health Organization	2016	Childhood Hearing Loss Strategies for prevention and care	Preschool– and school-aged children	/	/	Integrate ear and hearing screening in school health programs and develop links for provision of suitable medical, surgical, and rehabilitative care
3	America [8]	Hall JW	2016	Effective And Efficient Pre-School Hearing Screening: Essential For Successful Early Hearing Detection And Intervention (EHDI)	All pre-school children from the age of 6 months to 5 years	Include DPOAE, tympanometry, acoustic reflex for broadband noise signal, otoscopy, and pure tone hearing screening at 20 dB HL	**Six months to 4 years** Tympanometry, acoustic reflex for broadband noise signal, and otoscopy for secondary screening ** ≥ 4 years** Tympanometry, acoustic reflex for broadband noise signal, and otoscopy for children who do not pass DPOAE 2. Pure tone hearing screening at 20 dB HL for children who have normal tympanograms	DPOAEs as the primary tool for hearing screening of all pre-school children from the age of 6 months to 5 years
4	China [12]	National Health and Family Planning Commission of the People's Republic of China	2013	The Technical Specification for Children's Ear and Hearing Care	0–6 years of age	Include: Ear appearance examination, auditory behavioral observation, portable auditory assessment instrument, and OAE	**Referral for diagnosis:** 1. Positive results on any of the auditory behavioral observation method screening tools 2. Positive results on any of the audiological assessment instrument screening tools 3. Failure of OAE screening	1. After hearing screening in the neonatal period, children aged 0–6 years are managed in the health care system 2. Ear and hearing care is provided in conjunction with health screening 3. The priority ages for hearing screening are 6, 12, 24, and 36 months of age
5	Europe [35]	Skarżyński H et al	2012	Screening for hearing problems in pre-school and school-age children: European Consensus Statement	All children aged 4–7 years	/	/	1. Defining the role of pre-school and school screening programs in identifying and treating hearing problems; 2. Identifying the target population; 3. Recognizing the need for a quality control system in screening programs
6	America [11]	American Academy of Audiology	2011	Childhood Hearing Screening Guidelines	Pre-school; kindergarten; and grades 1, 3, 5 and either 7 or 9	Include: pure tone screening, tympanometry, acoustic reflex and reflectometry, screening with Speech Stimuli Materials and OAEs	1. Fail pure tone or OAE and tympanometry: Rescreening in 8–10 weeks 2. Fail pure tone only: no rescreening, do not wait for second-stage screening	**Pure tone screening:** 1. Perform a pure-tone sweep at 1000, 2000, and 4000 Hz at 20 dB HL 2. Present a tone more than once but not more than four times if a child fails to respond 3. Only screen in an acoustically appropriate screening environment 4. Failure is indicated by a lack of response at any frequency in either ear **Tympanometry screening:** 1. Employ a second‐stage screening method after failure of pure tone or OAEs 2. Use defined tympanometry screening and referral criteria 3. The target should be young children

#### General information of childhood hearing screening guidelines

We included six guidelines from six countries or organizations published between 2011 and 2017. Among them, one guideline was developed by the WHO, two by the American Academy of Audiology and Hall, and three by other expert groups, including England Audiology and Health, National Health and Family Planning Commission of the People's Republic of China, and Institute of Physiology and Pathology of Hearing in Poland.

#### Populations of childhood hearing screening guidelines

Table 4 indicates differences in the screening populations across the guidelines, which ranged from 0 to 9 years; however, most guidelines (2016 WHO, 2016 America, and 2013 China) recommend pediatric hearing screening for all preschoolers. Guidelines from England and Europe recommend screening children aged 4–7 years. Guidelines from the American Academy of Audiology recommend screening school-age children in pre-school; kindergarten; and grades 1, 3, 5, and either 7 or 9. China’s technical specifications recommend screening all children aged 0–6 years.

#### Screening technologies in childhood hearing screening guidelines

As shown in Table 4, 3 (50%) guidelines specify screening techniques and rescreening or referral processes while the remaining guidelines do not. Moreover, there were different screening technologies across the guidelines, including pure-tone hearing screening, OAE, tympanometry, acoustic reflex for broadband noise signal, otoscopy, speech stimuli materials, ear appearance examination, auditory behavioral observation, and portable auditory assessment instruments. China’s technical specifications recommend ear appearance examination, auditory behavioral observation, portable auditory assessment instruments, and OAE for childhood hearing screening.

### Quality assessment based on the AGREE II Score

Table 5 highlights the domain scores of guidelines according to AGREE II, including the score rates according to the domain, overall quality scores, whether the guideline is recommended, and ICC factors.

**Table 5:** Domain scores of the guidelines according to AGREE II

Guideline	Domain 1	Domain 2	Domain 3	Domain 4	Domain 5	Domain 6	Overall Score (mean)	Recommend this guideline for use			ICC (95%CI)	*F*	*P*
	Scope and purpose (%)	Stakeholder involvement (%)	Rigor of development (%)	Clarity of presentation (%)	Applicability (%)	Editorial independence (%)		Yes	Yes, with modifications	No			
**Newborn hearing screening**													
1.2019 America[19]	90.74	66.67	73.61	96.30	87.50	100.00	7	3	0	0	0.908 (0.832–0.955)	30.941	0.000
2.2018 Europe [20]	88.89	85.19	64.58	92.59	69.44	44.44	3	0	0	3	0.923 (0.855–0.963)	42.239	0.000
3.2018 Germany [21]	87.04	51.85	64.58	100	76.39	72.22	5	1	2	0	0.944 (0.895–0.973)	50.317	0.000
4.2018 South Africa [22]	83.33	70.37	72.22	96.30	95.83	27.78	6	3	0	0	0.869 (0.738–0.939)	26.254	0.000
5.2018ICON [23]	88.89	66.67	77.78	98.15	70.83	69.44	4.67	2	1	0	0.779 (0.616–0.888)	13.003	0.000
6.2017 India [24]	90.74	50.00	52.78	85.19	72.22	27.78	5	1	2	0	0.858 (0.747–0.929)	20.019	0.000
7.2017 Italy [25]	85.19	57.41	59.03	94.44	83.33	94.44	4.33	2	1	0	0.853 (0.739–0.926)	18.952	0.000
8.2016England [3]	90.74	62.96	72.22	100.00	80.56	44.44	6.67	3	0	0	0.856 (0.741–0.928)	20.337	0.000
9. 2016 New Zealand [26]	94.44	72.22	77.78	100.00	97.22	36.11	6	2	1	0	0.916 (0.845–0.959)	32.517	0.000
10.2016IPOG [27]	98.15	81.48	70.14	98.15	75.00	69.44	5.67	2	1	0	0.838 (0.693–0.922)	19.940	0.000
11.2013 Australia [28]	94.44	74.07	78.47	100.00	97.22	33.33	6.67	3	0	0	0.907 (0.831–0.955)	29.970	0.000
12.2011 Canada [29]	79.63	61.11	81.25	98.15	76.39	38.89	4.33	1	2	0	0.815(0.679–0.906)	14.631	0.000
13. 2010 China [30]	85.19	74.07	70.83	100.00	90.28	36.11	5	3	0	0	0.843 (0.691–0.910)	18.513	0.000
14.2010 CODEPEH [31]	83.33	72.22	70.83	10.00	94.44	61.11	5.33	3	0	0	0.872 (0.732–0.941)	28.226	0.000
15.2010 WHO [32]	90.74	74.07	83.33	96.30	97.22	66.67	5.33	2	1	0	0.911 (0.811–0.959)	40.722	0.000
Average	88.77	68.02	71.30	91.04	84.26	54.81	5.33						
**Childhood hearing screening**													
1.2017 England [33]	68.52	55.56	61.81	83.33	59.72	33.33	4	0	0	3	0.890 (0.801–0.946)	24.636	0.000
2.2016 WHO [34]	75.93	61.11	52.78	55.56	63.89	80.56	4	0	0	3	0.819 (0.682–0.909)	15.717	0.000
3. 2016 America [8]	87.04	51.85	79.86	100.00	70.83	41.67	5.33	3	0	0	0.843 (0.686–0.926)	22.044	0.000
4. 2013 China [12]	88.89	55.56	53.47	100.00	69.44	44.44	4.67	3	0	0	0.865 (0.758–0.933)	21.622	0.000
5. 2012 Europe [35]	90.74	51.85	60.42	88.89	59.72	47.22	4	1	2	0	0.840 (0.714–0.920)	18.364	0.000
6. 2011 America [11]	92.59	79.63	86.11	98.15	86.11	72.22	6.67	3	0	0	0.873 (0.765–0.938)	24.171	0.000
Average	83.95	59.26	65.74	87.66	68.29	53.24	4.78						

#### Guidelines for newborn hearing screening

The average scores for Domain 1, 2, 3, 4, 5, and 6 were 88.87% [range: 79.63% (2011 Canada) to 98.15% (2016 IPOG)], 68.02% [range: 50% (2017 India) to 85.19% (2018 Europe)], 71.30% [range: 52.78% (2017 India) to 83.33% (2010 WHO)], 91.04% [range: 10% (2010 CODEPEH) to 100% (2018 Germany, 2016 England, 2016 New Zealand, 2013 Australia, 2010 China)], 84.26% [range: 69.44% (2010 CODEPEH) to 97.22% (2016 New Zealand, 2013 Australia, 2010 WHO)], and 54.81% [range: 27.78% (2017 India, 2018 South Africa) to 100% (2019 America)], respectively. Overall, the mean score was 5.33 [range: 3 (2018 Europe) to 7 (2019 America)].

#### Guidelines for childhood hearing screening

The average scores for Domain 1, 2, 3, 4, 5, and 6 were 83.95% [range: 68.52% (2017 England) to 92.59% (2011 America)], 59.26% [range: 51.85% (2016 America) to 79.63% (2011 America)], 65.74% [range: 52.78% (2016 WHO) to 86.11% (2011 America)], 87.66% [range: 55.56% (2016 WHO) to 100% (2013 China, 2016 America)], 68.29% [range: 59.72% (2017 England) to 86.11% (2011 America)], and 53.24% [range: 33.33% (2017 England) to 80.56% (2011 America)], respectively. Overall, the mean score was 4.78 [range: 4 (2017 England, 2012 Europe, 2016 WHO) to 6.67 (2011 America).

#### Intraclass reliability

Table 5 presents the ICC for AGREE II for all the guidelines. We obtained a significant ICC (*P* < 0.05), which indicated a general consensus among the three reviewers. All guidelines achieved “excellent” intraclass reliability.

## Discussion

This study assessed the quality of global guidelines and consensus statements for newborn and childhood hearing screening programs; moreover, it analyzed and compared the characteristics of Chinese and international guidelines. Below, we discuss the general information regarding guidelines, screening principles, hearing screening protocols, hearing screening quality indicators, and quality assessment based on the AGREE II score.

### General information regarding guidelines

According to Morton’s study published in 2006, which was conducted in England where there is high compliance with confirmatory testing, permanent childhood hearing loss is defined as a bilateral sensorineural loss of ≥ 40 dB, with a reported incidence of 1.33 per 1000 newborns. The prevalence of permanent sensorineural hearing loss increases during childhood and approximately reaches 2.7 and 3.5 per 1000 children before the age of 5 years and during adolescence, respectively [36]. Hearing loss can affect communication, language, and speech development in children, cognition, education, employment, mental health, and interpersonal relationships. Hearing loss can cause low self-esteem, is often associated with stigma, and can adversely affect the families and communication partners of the patients [1].

Various countries and regional organizations are developing guidelines for newborn and childhood hearing screening. Chorath et al. identified and evaluated 12 newborn hearing screening guidelines in a systematic review [14]. Here, we included 15 newborn hearing screening guidelines from 15 countries or organizations published between 2010 and 2019. The total number of guidelines included in our study was three more than the those included in the study of Chorath et al., and nine guidelines were included in both. This is likely due to searching government and organizational websites in addition to searching databases. Among the guidelines, sixty percent required informed consent from parents, which indicates that it is a crucial aspect of newborn hearing screening.

We included six childhood hearing screening guidelines from five countries or organizations which were not included in the study of Chorath et al., with two being published by two American organizations [8, 11]. This suggests that compared with other countries, America has more guidelines on hearing screening for children and that its healthcare administrators may be more concerned about pediatric hearing screening. Childhood hearing screening guidelines were published between 2011 and 2017. There is a need to update these guidelines given the improvements in screening processes and technologies, as well as the accumulation of clinical experience.

### Screening principles and time

Given the varying recommendations of the six childhood hearing screening guidelines, only the newborn hearing screening principles are discussed here. Eight guidelines from America, Europe, ICON, Italy, New Zealand, Canada, CODEPEH, and WHO recommend the 1–3-6 principles, indicating international acceptance of this screening principle. Most guidelines recommend completing initial screening before discharge, with the exact timing varying based on the length of hospital stay in each country and region. Most guidelines recommend prompt rescreening of newborns who are not screened before discharge. Three guidelines cited different rescreening times, with the same diagnosis (within 3 months) and intervention times (within 6 months). Among them, the Chinese guidelines recommend that newborns who do not undergo initial screening should complete rescreening within 42 days after birth [30]. Since the growth and developmental health check-up is performed at the age of 42 days, hearing rescreening at 42 days could facilitate rescreening rates [37]. The Indian guidelines recommend complete rescreening within 4 weeks after the first screening or at 6 weeks during the first immunization visit [24], which could improve compliance with rescreening among newborns who fail the initial screening.

South African guideline recommends screening before 1 month after birth and within 6 weeks after birth; diagnosing within 1 month after discharge; and accepting interventions before and within 6 and 8 months after birth, respectively [22]. Moreover, the recommended screening and intervention times were slightly later than those in the 1–3-6 principles. However, the diagnosis and intervention times recommended by guidelines from England and Australia were earlier than those in the 1–3-6 principle. Guidelines from England recommend diagnosis within 4 weeks of screen completion and accepting intervention within 4 weeks of screen completion or the gestational age of 44 weeks [3]. Australian guidelines recommend diagnosis at 2 weeks after the first screening and within a corrected age of 3 months, as well as accepting interventions within 3 months and no later than 6 months of age [28].

Generally, the screening principle and time of newborn hearing screening recommended by Chinese guideline are consistent with international recommendations. Guidelines from several developed countries recommend diagnosis and intervention within 3 and 6 months, respectively. Contrastingly, guidelines from several developing countries recommend diagnosis and intervention within 1 and 6 months, respectively. This suggests that hearing screening principles should be developed according to the national context in terms of scientific validity and feasibility.

Regarding the follow-up time for newborns at risk, 2010 China guideline recommended that high-risk newborns should undergo annual follow-ups for 3 years even if they pass the initial hearing screening. Guidelines from America, South Africa, New Zealand, Australia, Italy, and CODEPEH recommended follow-up of high-risk newborns up to 9, 9, 18, 12, 12, and 24–30 months, respectively. Furthermore, 46.67% of the guidelines did not provide relevant information. The greater variability in the follow-up duration compared with the duration of initial screening, rescreening, diagnosis, and intervention indicates significant variations in the neonatal follow-up conditions across countries and regions.

### Hearing screening protocols

#### Newborn hearing screening

##### (1) Hearing screening process

Most guidelines recommend “initial screening-rescreening-diagnosis-intervention” for well-babies and “screening-diagnosis-intervention” for NICU newborns given the high rate of hearing loss among NICU newborns. The screening process for well-babies and NICU newborns in the Chinese guideline is consistent with mainstream international recommendations [30]. It is worth noting that the South African guideline recommended rescreening of NICU newborns who failed initial screening, which may be relevant to their specific national context.

##### (2) Screening technology

For well-babies, most guidelines recommended OAE for the first screening; however, 2018 Europe guideline only recommended AABR. Further, most guidelines recommended AABR for rescreening; however, those from South Africa and India recommended only OAE [22, 24]. This suggests that recommendations for screening technologies may be influenced by the level of economic development in individual countries and regions.

Chinese technical specifications recommend OAE or AABR for both initial screening and rescreening [30]. Wen et al. assessed the current status of the UNHS program at 26 institutions in China and reported that 61.54% and 73.08% of these organizations used OAE and OAE combined with AABR, respectively, for rescreening [38]. Taken together, recommendations regarding initial screening and rescreening technology in China are consistent with the mainstream international consensus; moreover, the current implementation status in China is consistent with the recommendations.

Compared with infants from well-baby nurseries, infants admitted to the NICU have a higher prevalence of increased hearing thresholds and a higher risk of auditory neuropathy [28]. Accordingly, most guidelines recommended AABR as a screening technology for NICU newborns to detect auditory neuropathy. Specifically, most guidelines recommend only AABR, guidelines from Italy and IPOG recommend combining OAE and AABR, and guidelines from CODEPEH combining OAE and AABR or using AABR only. Only Indian guidelines recommend the ABR test for NICU newborns to rule out auditory dyssynchrony/auditory neuropathy [24]. Moreover, Chinese technical specifications only recommend AABR for NICU newborns [30], which is consistent with the mainstream international opinion.

##### (3) Quality indicators

Eight and six guidelines mentioned initial screening coverage and the referral rate to diagnosis. Most of these guidelines recommended initial screening coverage of over 95% and a referral rate to diagnosis within 4%, indicating that these are common quality monitoring indicators for UNHS programs, and that agreements about screening sessions between those guidelines remain. However, guidelines from England, Australia, and Italy recommended initial screening coverage of > 97%, > 97%, and > 98%, respectively [3, 25, 28]. Guidelines from England and South Africa recommended a referral rate of > 2.5% (within 3%) and within 5%, respectively [3, 22]. The specific recommended values vary slightly across countries, with higher recommended values for initial screening coverage indicating a need to screen more newborns and a higher assessment requirement in hearing screening programs. Moreover, a higher referral rate to diagnostic audiological assessment could indicate more false positives in the screening test [4]. However, an extremely low referral rate may suggest that rescreening misses a proportion of newborns who fail the initial screening. Therefore, there is a need to set a minimum lower limit. The inconsistencies in initial screening coverage and referral rate reflect the actual situation of UNHS program across different countries and organizations. The inconsistencies may be explained by the fact that economically developed countries started UNHS programs earlier and demanded higher quality of newborn hearing screening.

Current Chinese guidelines do not mention the aforementioned screening quality indicators and updated guidelines should include them. According to Zhu and Li, in the past decade, China has made substantial prevention efforts by providing free services in poor areas, with newborn hearing screening increasing from 29.9% in 2008 to 86.5% in 2016 [39]. In 2020, we reported that an increase in the initial screening coverage from 94.96% in 2016 to 96.10% in 2017 [38]. Additionally, the referral rate to diagnostic audiological assessment in 26 Chinese institutions was 1.16% in 2016 and 1.24% in 2017 (both within 3%) [38]. This suggests generally good quality of newborn hearing screening in China; however, there may be regional differences. Therefore, there is a need to implement nationwide quality control measures for newborn hearing screening programs.

#### Childhood hearing screening

Hearing and genetic screening of 180,469 neonates with follow-up in Beijing, China, revealed that 25% of infants with pathogenic combinations of *GJB2* or *SLC26A4* variants and 99% of infants with an m.1555A > G or m.1494C > T variant passed routine newborn hearing screening, with subsequent presentation of delayed onset, progressive hearing loss, or susceptibility to ototoxic drugs [40]. Therefore, screening programs should be considered throughout pre-school ages. Taken together, childhood hearing screening is effective for early detection of late-onset hearing loss.

##### (1) Screening populations

There were among-guideline differences in the childhood hearing screening populations. Most guidelines recommend pediatric hearing screening for all preschoolers to facilitate early detection of new hearing loss and to maximize speech perception and attainment of linguistics-based skills. The American Academy of Audiology has issued broad guidelines for screening childhood hearing, including preschoolers, as well as grades 1, 3, 5, and either 7 or 9, to identify approximately 70% of previously unidentified hearing losses [11]. The technical specifications in China stipulated that after the newborn hearing screening, children aged 0–6 years should be managed in the health care system. Moreover, ear and hearing care should be provided at the same time as the health checks, with the priority ages for hearing screening being 6, 12, 24, and 36 months, which are feasible follow-up time points for child health management [12, 37].

##### (2) Screening technology

There were among-guideline differences in the recommended screening techniques for children screening. The American Academy of Audiology recommends that young children should be targeted for tympanometry screening and that children aged ≥ 3 years (chronologically and developmentally) undergo pure tone screening. Moreover, the results of pure tone or OAE and tympanometry rescreening should inform subsequent steps [11]. The American guidelines of 2016 considered OAE as the primary hearing screening tool of all pre-school children aged 6 months to 5 years [8]. For children aged from 6 months to 4 years, the secondary screening techniques are tympanometry, acoustic reflex for broadband noise signal as indicated, and otoscopy as indicated. For children aged ≥ 4 years, rescreening involves pure-tone hearing screening at 20 dB HL [8]. In summary, both American guidelines have age-specific recommendations for screening techniques.

Similarly, the technical specifications of China have age-specific recommendations. Auditory behavioral observations are recommended for children aged < 3 years, with this population being divided into the following age groups: 6 months, 12 months, 24 months, and 36 months. Moreover, there are age-specific recommendations for hearing screening using portable auditory assessment instruments; specifically, a 60 dB SPL sound at a 2-kHz warble tone for children aged < 12 months, a 55 dB SPL sound field at 2-kHz and 4-kHz warble tones for children aged 12–24 months, and use of headphones or pure tone screening at a 45 dB HL sound at 1, 2, and 4 kHz for children aged 3–6 years [12]. Additionally, equipped community and township health centers can perform hearing screening using screening otoacoustic emission devices [12].

Only 50% of the guidelines specify screening techniques and rescreening or referral processes, suggesting a need to develop hearing screening guidelines for children and improve them to allow large-scale and systematic implementation of hearing screening for children.

### Quality assessment based on the AGREE II Score

The quality of the guidelines was determined based on the average scores for the six domains and the overall scores for each guideline. Chorath et al. reported ICC analysis of 12 guidelines showed good to very good agreement across all domains [14]. We included 21 guidelines with ICC ≥ 0.75, indicating a high degree of consistency and confidence in the scores, which was consistent with the above study.

#### (1) Newborn hearing screening

Among newborn hearing screening guidelines, the score hierarchy of the domains in descending order was as follows: ‘Clarity of Presentation’ domain (91.04%), ‘Scope and Purpose’ domain (88.87%), ‘Applicability’ domain (84.26%), ‘Rigor of Development’ domain (71.30%), ‘Stakeholder Involvement’ domain (68.02%), and ‘Editorial Independence’ domain (54.81%). Similarly, in a systematic evaluation of clinical practice guidelines on newborn hearing screening, the ‘Scope and Purpose’ domain achieved the highest mean score (91.30%) [14]. The score of ‘Scope and Purpose’ domain in our research was close to 91.3%, indicating detailed descriptions regarding scope and purpose. However, the lowest score (35.80%) of ‘Rigor of Development’ was much lower than 71.30%, possibly due to differences in included guidelines, indicating unclear descriptions regarding rigor of development. Additionally, there may be unclear descriptions regarding editorial independence and stakeholder involvement.

The American guideline had the highest overall score, which was consistent with the systematic quality appraisal in 2021 [14]. This could be attributed to American hearing screening guidelines being developed following the principles of evidence-based medicine; having recommendations supported by good evidence; and referring to detailed and comprehensive information regarding hearing screening, referral, and intervention. Additionally, the American guidelines are more consistent with the requirements of the AGREE II instrument, present specific recommendations, clearly present different management alternatives for the health issues, and describe factors that facilitate and limit implementation. This position statement reflects the views and opinions of the authors and does not necessarily reflect the official policy or position of the member organizations, as highlighted in the acknowledgments [19]. The European guideline had the lowest overall score, which could be attributed to a lack of details regarding the applicability and editorial independence. Although European guidelines have the advantage of grading evidence for recommendations, there are focused on the responsibilities of parents and family, healthcare professionals, neonatal units, hospitals, follow-up teams, and health services, without mentioning the quality indicators for hearing screening.

For the Chinese guideline included for quality assessment for the first time, the hierarchy of the domain scores were as follows: 100.00% (domain 4),90.28% (domain 5), 85.19% (domain 1), 74.07% (domain 2), 70.83% (domain 3), and 36.11% (domain 6); moreover, the overall score was 5. Overall, the technical specifications for hearing screening in China have good quality; however, content regarding stakeholder involvement, rigor of development, and editorial independence could be further improved.

#### (2) Childhood hearing screening

Among the childhood hearing screening guidelines, the score hierarchy of the six domains was as follows: domains 4, 1, 5, 3, 2, and 6. Notably, all six scores were lower than those of newborn hearing screening guidelines, suggesting that childhood hearing screening guidelines have lower overall quality than newborn ones. An American guideline (2011 America) had the highest overall score, which can be attributed to authoritative recommendations, evidence-backed screening techniques, comprehensive screening populations, and detailed screening and referral protocols. Guidelines from England, Europe, and the WHO had the lowest overall scores since they did not reflect systematic screening and referral protocols.

The Chinese technical specifications had the third-highest score after the two American guidelines and recommended detailed screening populations, screening techniques, and referral protocols, with good overall quality, implement ability, and generalizability. However, there were low scores of stakeholder involvement, rigor of development, and editorial independence (55.56%, 53.47%, and 44.44%, respectively), which suggests that these areas could be further improved in updated guidelines.

### Strengths and limitations

The guidelines represent the development of hearing screening for newborns and children in each country. Moreover, information regarding hearing screening reflects the actual situation in each country or organization, as well as the theoretical consensus and practical problems regarding guideline implementation. Therefore, it is necessary to compare information regarding screening to confirm consistency between Chinese guidelines and those of other countries and organizations, as well as to identify more accurate processes for hearing screening, which could provide a scientific basis for the revision of the guidelines in China. Moreover, this study indicates the requirement to update guidelines based on evidence to ensure evidence-based best practice and standardized implementation of hearing screening and higher quality screening. The limitation of this study is that due to the language limitation, we were not able to analyze the guidelines or consensus statement other than English and Chinese.

## Conclusion

In conclusion, our systematic review highlighted that newborn hearing screening guidelines are of better quality than childhood guidelines; this may be due to the fact that newborn hearing screening was conducted earlier and more widely than pediatric hearing screening. Both the 2019 American position statement and the 2011 Childhood Hearing Screening Guidelines demonstrated superior quality due to comprehensive expert teams, regular updates to guidelines, and evidence-backed recommendations. Comparative analysis suggested that recommendations of the Chinese newborn and pediatric hearing screening protocols are consistent with mainstream international opinion. Moreover, this analysis demonstrated that ‘Editorial Independence’ and ‘Stakeholder Involvement’ have the greatest opportunities for improvement. These results may help to progress the quality of hearing screening guidelines in clinical practice and guide evidence-based updates.

## Data Availability

All data generated or analysed during this study are included in this published article.
